# Bridging Cultures and Generations in the Caregiving of Midlife Chinese Immigrant Women

**DOI:** 10.1093/geroni/igaf122.1892

**Published:** 2025-12-31

**Authors:** Bo Jian, Merril Silverstein, Catherine Garcia

**Affiliations:** Syracuse University, Syracuse, New York, United States; Syracuse University, Syracuse, New York, United States; Syracuse University, Syracuse, New York, United States

## Abstract

This study explores the well-being of midlife Chinese immigrant women as they navigate the complexities of intergenerational caregiving within the intersecting contexts of immigration, family obligations, and aging. Specifically, it examines how these women adapt to dual caregiving roles—supporting their children’s education while caring for aging parents—amid the constraints of their immigrant experience. Using an ethnographic approach, this study draws on in-depth interviews and field notes from 20 immigrant Chinese women aged 35–50, analyzed through reflexive thematic analysis. This qualitative methodology captures the nuanced experiences of caregiving across transnational contexts, highlighting how cultural expectations, economic precarity, and shifting caregiving norms shape midlife immigrant women’s daily lives. Findings reveal that caregiving responsibilities contribute to significant role strain, stress, and health consequences, including anxiety, emotional distress, premature hair graying, breast conditions, and migraines. Many participants prioritize their children’s academic success over their own aspirations, further intensifying their caregiving burden. To cope, they turn to ethnic community networks, adopt flexible caregiving strategies, and advocate for culturally responsive resources. However, structural barriers such as language access, employment precarity, and policy inaccessibility persist, limiting their ability to seek formal support. This study underscores the urgent need for culturally inclusive policies and community-based interventions that recognize the intersection of gender, caregiving, and immigration. Addressing these challenges can help midlife immigrant women balance caregiving responsibilities while safeguarding their well-being.

